# Siland a R package for estimating the spatial influence of landscape

**DOI:** 10.1038/s41598-021-86900-0

**Published:** 2021-04-05

**Authors:** Florence Carpentier, Olivier Martin

**Affiliations:** 1 Université Paris-Saclay, INRAE, UR MaIAGE, 78350 Jouy-en-Josas, France; 2 AgroParisTech, 75005 Paris, France; 3 INRAE, BioSP, 84914 Avignon, France

**Keywords:** Agroecology, Ecological modelling

## Abstract

The spatial distributions of populations are both influenced by local variables and by characteristics of surrounding landscapes. Understanding how landscape features spatially structure the frequency of a trait in a population, the abundance of a species or the species’ richness remains difficult specially because the spatial scale effects of the landscape variables are unknown. Various methods have been proposed but their results are not easily comparable. Here, we introduce “siland”, a general method for analyzing the effect of landscape features. Based on a sequential procedure of maximum likelihood estimation, it simultaneously estimates the spatial scales and intensities of landscape variable effects. It does not require any information about the scale of effect. It integrates two landscape effects models: one is based on focal sample site (*Bsiland,* b for buffer) and one is distance weighted using Spatial Influence Function (*Fsiland*, f for function). We implemented “siland” in the adaptable and user-friendly R eponym package. It performs landscape analysis on georeferenced point observations (described in a Geographic Information System shapefile format) and allows for effects tests, effects maps and models comparison. We illustrated its use on a real dataset by the study of a crop pest (codling moth densities).

## Introduction

Numerous studies demonstrate that the distribution of species richness and abundance depend on both local and landscape variables^1–3^. However, studying the relationships between landscape and species distributions remains challenging because the shape and the scale of landscape effects are unknown^4^ and can be missed if assessed at an incorrect scale^5^. The studied data usually contains georeferenced observations at point sites, named response variables hereafter, and the description of several landscape spatial variables, named landscape variables hereafter. Their studies are often referred to as focal patch studies^6^. To identify the scale of landscape variable effects, the common approach consists of the following: (i) a priori defining a set of scales; (ii) creating summary variables by computing measures of the landscape variables within discs or rings of radii equal to each scale centered on the observation sites (named buffers hereafter); and (iii) applying a regression model to the response variable with the summary variables as the explanatory variables, for example, a linear model or a random forest algorithm^7^. For each landscape variable, the scale of effect is then considered to be the size of the buffer best explaining the response variable.

The main disadvantage of this method is that the number of explanatory variables artificially increases with the number of spatial scales considered. One then faces a complex statistical dilemma, which is dealing with numerous explanatory variables that by their construction are highly correlated. Consequently, the potential scales chosen are often too few and their ranges are too limited^8^. Finally, the effect of a landscape variable is modelled as uniform within the buffer and as null outside it^9^, which is unrealistic and biologically unjustified as a continuously decreasing effect is expected^10^. Several new methods based on distance-weighted effects have been proposed to model a distance-decreasing effect^11–13^, but they explore a limited predefined set of spatial scales for predictors. Other methods quantified the scale of landscape effects without an a priori choice of spatial scales^9,14^. However, none of these methods are yet implemented in a ready-to-use software. Huais^15^ proposed a very convenient R function “multifit” to select scales but with some limitation (it is not distance weighted, requires the choice of a set of scales), while calling for further developments of such a method to generalise this type of automated analysis. At present the analysis of landscape effects on population parameters (e.g. abundance or occurrence) therefore remains complex and the results obtained with the methods mentioned above are not easily comparable.

Here we present a general method for landscape effect analysis. We implemented this method in “siland”, a package for the R statistical computing environment. Based on a sequential maximum likelihood estimation procedure, our method improves current methods by providing the joint estimation of the effects of landscape variables as well as the spatial scales of these effects and by allowing comparisons between two spatial effects models. In the first model, Bsiland method hereafter (B for buffer), the effect of a landscape variable is modeled as in classic methods based on focal sample site, i.e. considered as constant over a disc centered on the observation point. But contrarily to the previous method, it does not require a first definition of tested radii since the optimal buffer radii are estimated. In the second model the effect of landscape is based on a weighted distance, as in the framework proposed by Chandler^9^. The decrease in weight with distance is modeled by a Spatial Influence Function (SIF). The parameter of the SIF defined the scale of effect of a landscape variable. In this second method, named Fsiland (F for function), the parameters of the SIFs are estimated for each landscape variable.

The main functions of siland allow the user (i) to estimate the intensity of local and landscape effects and the scale parameter of each landscape variable, (ii) to test these effects and (iii) to plot landscape effects on maps. We exemplified the method and the package use by analysing the landscape effects of conventional and organic orchards on the density of codling moth larvae per apple tree.

## Results

### Package description

The siland package is written entirely in the scientific computing language R^16^. It is available on CRAN (https://cran.r-project.org/package=siland). The analyses presented here were performed using the package siland 2.0.4.

### Case study

**Figure 1:**
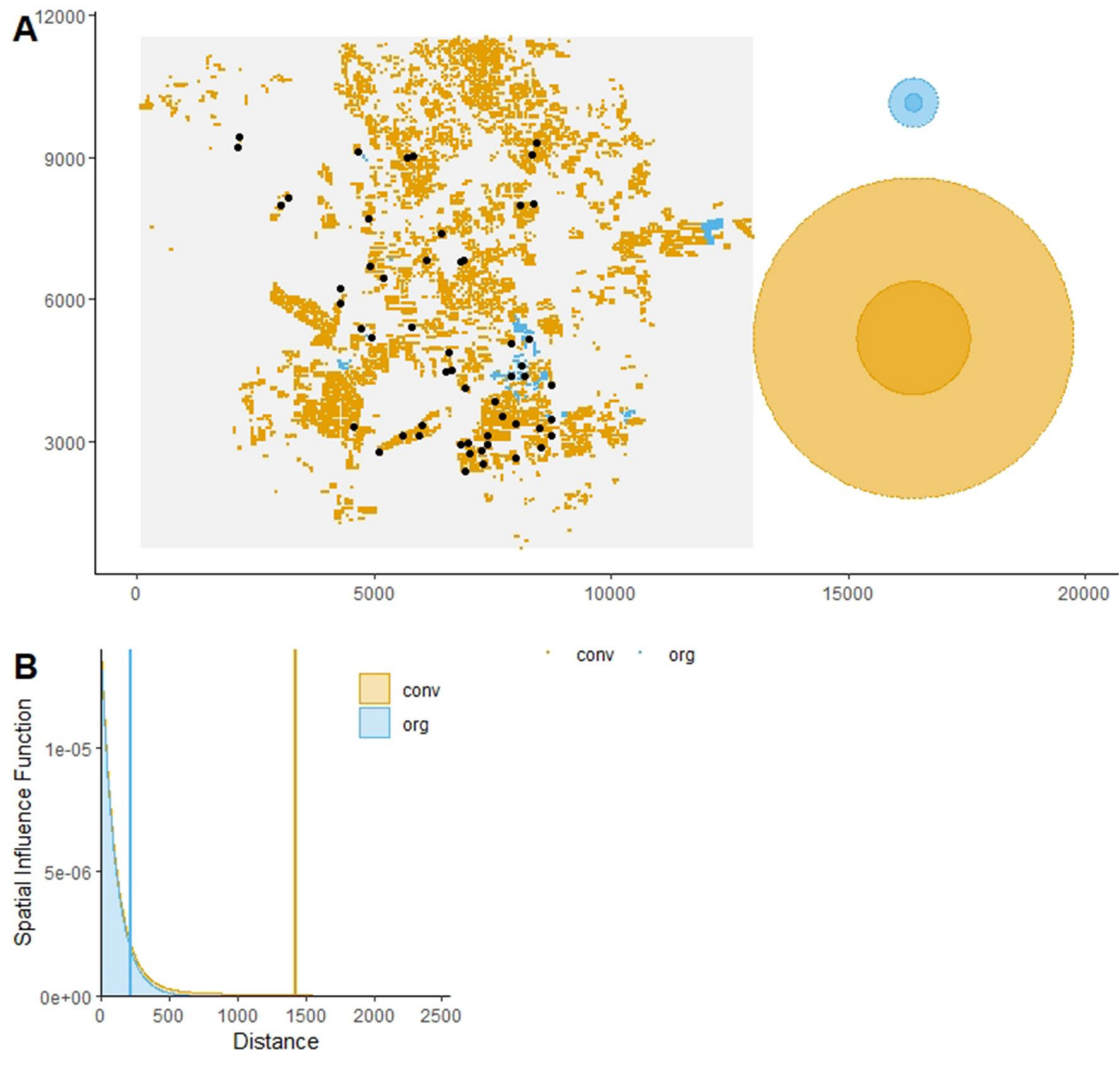
Map of the observations in the study site and the estimated spatial influence functions (SIFs). The figure A is obtained with plotFsiland(resF,landCmoth,data = dataCmoth). Black points represent locations of response variable observations. Yellow and blue squares are the pixels where conventional and organic orchards respectively are present. At the right margin, the light and dark discs represent area of medium influence and significant influence, respectively. Blue and yellow discs represent conventional and the organic orchards, respectively. The figure B is obtained with plotFsiland.sif(resF). The blue and orange lines represent the estimated SIF for the organic and conventional orchards, respectively. The vertical lines represent the SIF mean.

We illustrated the abilities of siland on an example previously described and analyzed in Ricci^17^: the study of codling moth densities, an insect pest specialized on apple orchards in the Basse Durance Valley in southeastern France (see Fig. 1). The datasets can be extracted from the package using the commands data(dataCmoth) and data(landCmoth). The complete analysis script and outputs are available in Supplementary Information file 1 (SI 1). dataCmoth is a data frame with two columns named X and Y containing the observation locations, a column Cmoth, containing the response variable of the study (the mean number of codling moths in the orchards), and a column trait, describing a local variable (the number of insecticide treatments applied in the orchard). landCmoth is a sf object (from package sf^18^) describing the landscape variables: conventional tree orchards (conv), organic tree orchards (org) where conv is equal to 1 if the land use is associated to orchard with conventional practice and 0 otherwise, and so is it for org.

### Main functions

The main functions are described in Table 1, while a full description of package functions is available at https://cran.r-project.org/web/packages/siland/siland.pdf. The package siland requires two objects containing data. The first object is a data frame composed by two columns named X and Y containing the observation locations, a third column representing the response variable and eventually other columns representing local variables. The second object is a sf object describing landscape variables. It can be obtained directly from shape files of landscape map by using the function st_read() of the package sf (see vignette(“siland") for more details).

**Table 1:** Main functions of siland package. Detailed information about these functions are given in https://cran.r-project.org/web/packages/siland/siland.pdf.

Functions for buffer approach	Functions for approach with SIF	Description
Bsiland	Fsiland	Estimation of model parameters for local and landscape variables and for scale of landscape effects
Bsiland.lik	Fsiland.lik	Graphical representation of profiled likelihood function of scales of effect of landscape variables
plotBsiland.land	plotFsiland.land	Graphical representation of landscape effects
	plotFsiland.sif	Graphical representation of the spatial influence functions
summary		Summary gives estimates and values for tests (similar to summary.lm and summary.glm)
AIC		AIC value of the estimated model
BIC		BIC value of the estimated model
residuals		Vector of residual values for the estimated model
fitted		Fitted values of the estimated model

Model estimation is performed using the function Bsiland() for Bsiland method and the function Fsiland() for Fsiland method. The arguments of the both functions are similar: formula of the model, land the sf object describing landscape variables and data, the observation data frame. The syntax of the formula “y ~ model” is similar to lm() function of the stat package. In the model term of the formula, the explanatory variables are added using the symbol “+”. The explanatory variables described in the data frame data are considered as local in the model, those described in the sf object land are considered as landscape variables. Local effects can be modelled as fixed or random (using the syntax (1|x), see vignette(“siland”) for more details). Interaction terms can be considered using the usual symbols “*” or “:”. Notice that only interactions between local x local and local x landscape variables are considered. Various types of response variables can be considered using the argument family which describes the assumed distribution of the response variable and can take the values "gaussian", "poisson" and “binomial” for data of continuous, counting or presence-absence types, respectively (and their associated link functions identity, log and logit respectively). 

**Figure 2:**
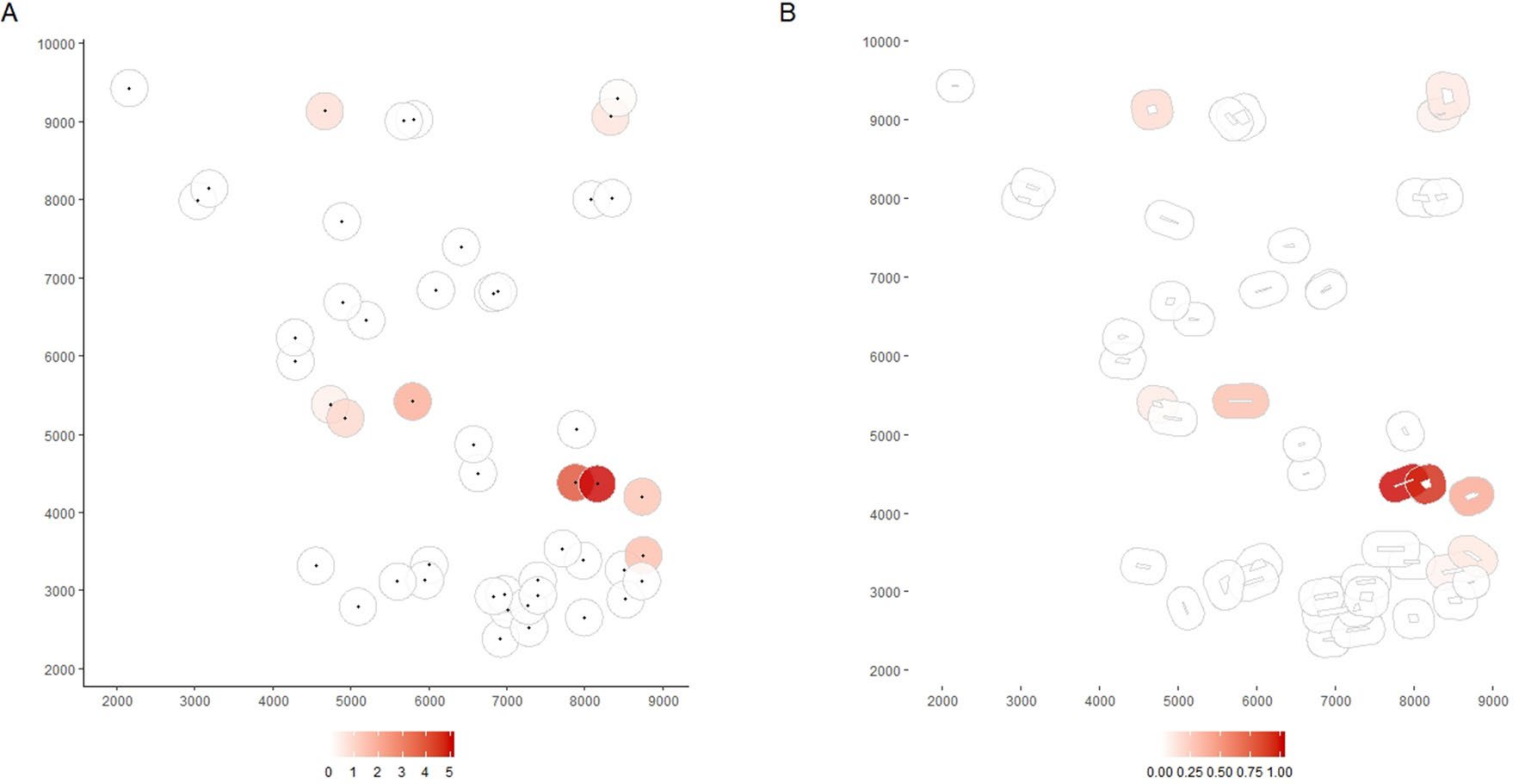
Maps of the predicted effect estimated by the Bsiland method obtained with the function plotBsiland.land(). The buffer model was estimated using the command: resB2 = Bsiland(Cmoth ~ trait + conv + org,land = landCmoth,data = dataCmoth,border = border). The colored area represents the buffer around each observation location estimated for the organic orchards effect. At the bottom margin, the bar color gives the effect intensity. Buffers are modeled in graphic A, from the observation locations (border = FALSE) and in graphic B from the border of the orchard of each observation(border = TRUE).

Using the argument border, the spatial effect of landscape variable can be considered from the observation locations (border = F) or from the border of the polygon where observations are located (border = T) (see Fig. 2). For Fsiland(), the additional argument sif indicates the family of SIF chosen ”exponential” (by default), “gaussian” or “uniform”.

The object returned by the functions Bsiland() and Fsiland() displays the parameters estimation and a test of the global landscape effect. The function summary() applied on the result object provides significance tests of the intensity of the effect of explanatory variables (local or landscape).

The two methods are based on likelihood maximization. The functions Bsiland.lik() and Fsiland.lik() aim to point out some optimization problems. They provide representations of the minus loglikelihood in function of buffer radius or mean distance of the SIF, respectively (see SI Fig. 1). Values of minus loglikelihood lower than the estimated one indicate that the estimation procedure did not perform correctly. In such cases, the estimation needs to be reiterated using different initial values of effects scales (with argument init of functions Fsiland() and Bsiland()). 

### Graphics outputs

**Figure 3:**
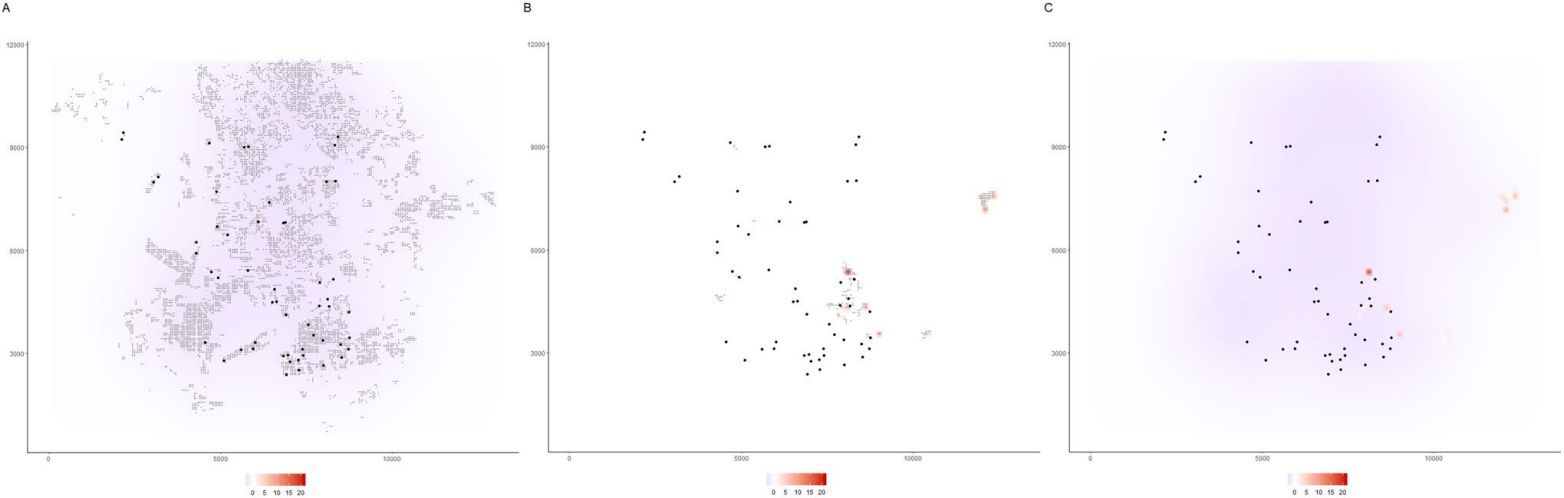
Maps of the predicted effect estimated by the Fsiland method obtained with the function plotFsiland.land(). The SIF model was estimated using the command: resF = Fsiland(Cmoth ~ trait + conv + org, land = landCmoth,data = dataCmoth). The three maps A, B and C were obtained with the command plotFsiland.land(resF, land = landCmoth, data = dataCmoth, var = var), var equal to 1,2 and 0 respectively. The black points represent locations of response variable observations. In graphics A and B, the gray points are the pixels where conventional and organic orchards are present, respectively. The response surface represents the cumulative effect of the conventional orchards in graphic A, the organic orchards in graphic B and the global landscape in graphic C. At the bottom margin, the bar color gives the effect intensity. In the figure A, conventional orchards had negative effects at large scale. In figure B, organic orchards had positive effects positive effects at small scale. In figure C, global landscape had an overall negative effect except on spotty areas.

The package siland proposes various functions for graphic representation of estimated landscape effects. The function “plotFsiland.land” displays landscape effect map for the estimations of the Fsiland method (Fig. 3). The influence of each landscape variable is plotted over all the study area. The global influence of the landscape, i.e. the sum of the effects of all landscape variables can also be plotted. The function plotBsiland.land() displays landscape effect map for the estimation of the Bsiland method (Fig. 2). For each landscape variable, the estimated area of effect (buffer of estimated radius) is plotted around each observation locations. The color of the area represents the estimated intensity of the effect i.e. the effect value multiplied by the proportion of the landscape variable in the area of effect.

#### Interpreting spatial influence functions (SIF)

The SIF function describes how the influence of a pixel/cell of a landscape variable is spatially distributed. We assume that the influence is maximal at the pixel location and decreases with the distance. The greater the estimated mean distance of the SIF, the greater the scale of effect of the landscape variable. The estimated SIF can be displayed using the function plotFsiland.sif() (see Fig. 1). The function Fsiland.quantile() allows to quantify the area of medium influence and significant influence of a landscape variable, that we defined as the disc containing 50% and 95% of the influence of the SIF (neglecting 50% and 5% of its broader effect) respectively. They can be compared to the landscape variables distribution in the study area using function plotFsiland() (see Fig. 1). Note that a Fsiland model with an uniform SIF is similar to Bsiland model. However, in siland package the Bsiland() function calculates the exact percentage of the landscape variable in buffers, when Fsiland() is based on an approximation, i.e. the rasterization of the landscape. As a consequence, the estimation with Fsiland is often faster than with Bsiland but may be erroneous if the definition of the raster is not precise enough. 

## Conclusion

Estimating scales of effect of landscape variables currently remains a great challenge, and consequently so does the estimation of landscape effect. So far no common methodology has emerged. With the siland package, we propose a tool that we believe is useful for all landscape ecologists who wish to investigate this type of question. Here, we have illustrated how with only few siland functions and limited knowledge of R, it is possible to conduct a reproducible and detailed study of multi-scale landscape effect including estimations but also tests and graphs illustrating the results obtained. The “siland” package is very adaptable, it integrates two methods and can handle various types of data (continuous, binary, discrete..). Applications of siland in a multiyear or multisite framework are already available for the buffers method and presented in the vignette(“siland”). However future developments are still needed to handle the increasing complexity of questions and data (numerous sites, years and dynamic data). 

## Models and methods

We consider a response variable measured at *n* different sites denoted *Y*_*i*_ (*i* stands for a site), *L* local variables which can be continuous or discrete and are denoted as *x*_*i*_^*l*^ (*l* stands for a local variable and *i* for a site) and *K* landscape variables denoted as *z*_*r*_^*k*^ (*k* stands for a landscape variable and *r* for a polygon in the landscape). In the Bsiland method, the effect of landscape variables is modelled using buffers with $p_{{i},\delta_{k}}^{k}$, the percentage of the landscape variable *k* in a buffer of radius *δ*_*k*_ and centered on site *i*. Since the Bsiland model is based on the generalized linear models framework, the expected value of the response variable *Y*_*i*_ is modelled as follows:

1 $$ \mu_{i} = \mu + \sum\limits_{l \in L} {\alpha_{l} x_{i}^{l} } + \sum\limits_{k \in K} {\beta_{k} p_{{i},\delta_{k}}^{k} } $$

where *µ* is the intercept, α_l_ and β_k_ are the effects of local and landscape variables, respectively.

The Fsiland method is based on Spatial Influence Functions (SIFs) in a similar framework to Chandler & Hepinstall-Cymerman ^9^. To simplify computations, the entire study area is not considered as continuous but rasterized, *i.e.* pixelated on a regular grid, named *R.* The value of each landscape variable *k* at a pixel *r* is described in *z*_*r*_^*k*^. For instance, if the landscape variable *k* is a presence/absence variable, *z*_*r*_^*k*^ is equal to one or zero. The expected value of the response variable *Y*_*i*_ is then modelled as follows:

2 $$ \mu_{i} = \mu + \sum\limits_{l \in L} {\alpha_{l} x_{i}^{l} } + \sum\limits_{k \in K} {\beta_{k} } \sum\limits_{r \in R} {f_{{\delta}_{k}} (d_{i,r} )z_{r}^{k} } $$

where *f*_*δk*_(*.*) is the **SIF** associated with the landscape variable *k* and *d*_*i,r*_ is the distance between the center of pixel *r* and the observation at site *i*. The SIF is a density function decreasing with the distance. The scale of effect of a landscape variable *k* is calibrated through the parameter δ_*k*,_ the mean distance of *f*_*δk*_. Two families of SIF are currently implemented in the siland package, exponential and Gaussian families defined as *f*_*δ*_(*d*) = 2*/*(*πδ*^2^)*exp*(*-*2*d/δ*) and *f*_*δ*_(*d*) = 1*/*(2*δ*)^2^*exp*(*-π*(*d/*2*δ*)^2^), respectively^19^. The effect of a landscape variable *k* is modelled by two parameters: an intensity parameter, *β*_*k*_ describing its strength and its direction and a scale parameter, *δ*_*k*,_ describing how this effect declines with distance. Each pixel potentially has an effect on the response variable at any observation site. No set of scales of effects is initially determined. In Eq. 2, the sum on the regular grid R is an approximation of the integration on the continuous study area. The choice of the grid definition is a tradeoff between computing precision and computing time. The smallest the mesh size of the grid is, the better are the precision but the longer the computing time is (and the larger the required memory size is). The parameters estimation may be very sensitive to this mesh size. To obtain a reliable estimation, we recommend to ensure, after the estimation procedure, that mesh size is at least three times smaller than the smallest estimated SIF (see Supplementary Fig. S2 online for details). If not, it is recommended to proceed with a new estimation with a smaller mesh (by using the wd argument of the Fsiland function, set at 30 by default).

All parameters, *µ*, {α_1_,…, α_*K*_}_,_ {β_1_,…, β_*K*_} but also {*δ*_1_,…, *δ*_*K*_} are simultaneously estimated by likelihood maximization for both Bsiland and Fsiland methods. We have developed a sequential algorithm. At the initialization stage, values are arbitrarily defined for the {*δ*_1*,..*_*,δ*_*K*_} scales parameters. In step A, the *µ*, {α_1_,.., α_*K*_}_,_ {β_1_,.., β_*K*_} parameters are estimated using the classical maximization procedures implemented in the lm and glm functions knowing the fixed values of the scale parameters. In step B, the scale parameters are estimated by likelihood maximization knowing the parameters estimated in step A. The values of the scale parameters are then fixed at the new estimated values. Steps A and B are thus repeated until the relative increase in likelihood decreased below a threshold or the maximum number of repetitions is reached. Tests performed (obtained using the summary function) are similar to those given by summary.lm or summary.glm function (see R Core Team^16^ for details, this implies that tests are given conditionally to the estimated scale parameters.).

## Supplementary Information

Supplementary Information 1.

Supplementary Information 2.
