# Supplementary material for: Siland a R package for estimating the spatial influence of landscape
Source: Sci Rep. 2021 Apr 5;11:7488. doi: 10.1038/s41598-021-86900-0 (PMC8021544; doi:10.1038/s41598-021-86900-0)

# **Siland a R package for estimating the spatial influence of landscape**

# **Supplementary Information Figure S2**

**Error rate as a function of mesh size (wd).** In the Fsiland method, the influence of a landscape is modeled as the integral of the spatial influence function (SIF) on the continuous study area. The calculation of this integral is approximated a sum on a regular grid of mesh size wd. The smallest the mesh size of the grid is, the better are the precision (but the longer the computing time is). We represented here the absolute error rate of this approximation (|approximated value - true value|/true value) calculated in the worst case : a variable present uniformly over the entire area, for 3 exponential SIF with mean distances, δ=50 (black line), 100 (red),200 (blue) and 300 (green)**.** The vertical dotted lines represent mesh sizes equal to δ/3. When the mesh size is less than δ/3, the approximation error remains less than 1%.


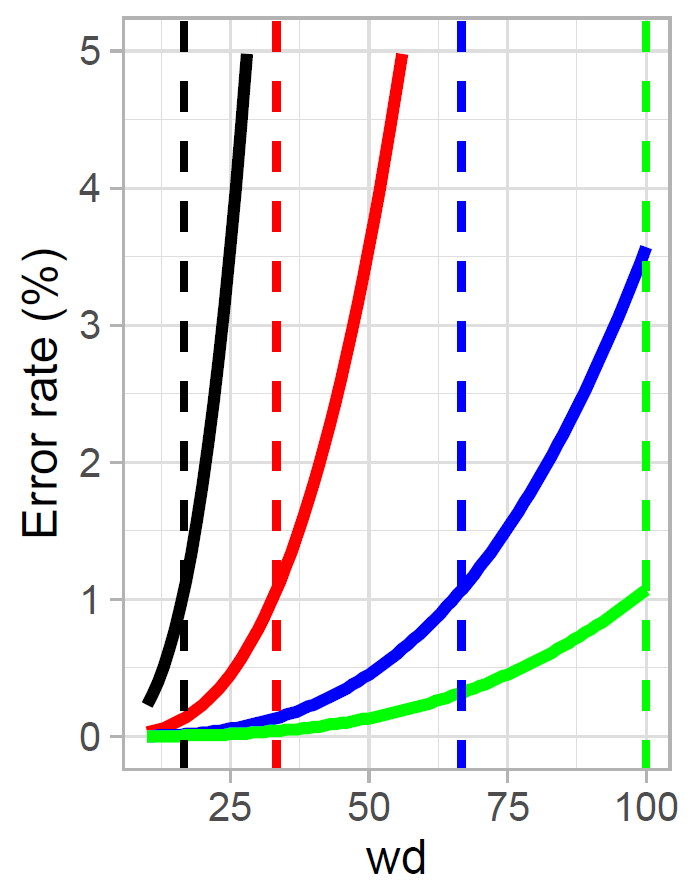

Supplement: Supplementary file 1 — Supplementary Information 1. [file 41598_2021_86900_MOESM1_ESM.docx]
